# Reports of Cases

**Published:** 1895-04

**Authors:** 


### REPORTS OF CASES.

---

A. J. Harrison, M.B., President of the Bristol Medico-Chirurgical Society, in a recent address before that Society, referring to his experiences in the Clifton Zoological Gardens, related an account of the sudden death during the night of the finest and apparently healthiest lion of the gardens up until two months before. Autopsy revealed an enormous internal hemorrhage from the spleen, which organ had probably ruptured during the exertion of coitus. He looked upon the splenic enlargement as arising from hyperæmia and increase in the lymphatic and vascular elements, but could only conjecture as to etiology. He asks the question, "Are lions subject to malarial attacks?" When first noticed ill his breathing was much disturbed, as in pneumonia.

Another case of ingrowing nails, demanding extraction, was finally accomplished by the use of the "Cramp cage," where, by limiting the space in which he could move, the paw was pushed out and a dextrous movement enabled seizure of the claw with a pair of carpenter's pincers and, aided by the vigorous movements of the animal, extraction was accomplished. The animal, a half-hour later, seemed to appreciate the result accomplished and became perfectly calm.

A third case of much interest in a lion but four-and-a-half months old, found dead in its cage after ailing for three or four days, with distressed breathing and loss of appetite and but little desire for water, exhibited on post-mortem distention of the pericardium, with a semi-purulent fluid of the consistency of gruel tinged with blood. Dr. Harrison considers lions short-lived when kept in captivity. One dying at sixteen years of age he considered due to senile decay.

The death of a lioness during parturition from rupture of the right horn of the uterus after being in labor for five days. One cub had been born, the other engaged in the passage.

